# Mortality Rates from Suicide in Brazil in 2021: A Comprehensive Demographic Analysis by Sex and Age Group

**DOI:** 10.1192/j.eurpsy.2025.1493

**Published:** 2025-08-26

**Authors:** J. R. Pinto Nasr

**Affiliations:** 1 Universidade Paulista - Campus Campinas, Campinas, Brazil

## Abstract

**Introduction:**

Suicide represents a significant and growing public health challenge in Brazil, reflecting a complex interplay of social, economic, and mental health factors.

The increasing rates of suicide highlight the need for targeted interventions and policies. Understanding the demographic characteristics associated with suicide, particularly in relation to sex and age, is crucial for developing effective prevention strategies and health policies. This study utilizes data from the 2024 epidemiological bulletin, “Panorama dos Suicídios e Lesões Autoprovocadas no Brasil de 2010 a 2021.”

**Objectives:**

This study aims to provide an analysis of the mortality rates from suicide in Brazil for the year 2021. The primary focus is on exploring the distribution of suicide rates by sex and age group, as well as evaluating the proportional mortality in relation to the total number of deaths in the country.

**Methods:**

The study utilized data sourced from the Mortality Information System (SIM) and the aforementioned epidemiological bulletin, which compiles comprehensive mortality data across Brazil. We analyzed the rates of mortality from suicide, categorizing the data by age groups: 05 to 14 years, 15 to 19 years, 20 to 29 years, 30 to 49 years, 50 to 69 years, and 70 years and older. The analysis further differentiated the data by sex, allowing for a nuanced understanding of demographic variations.

**Results:**

In 2021, Brazil reported a total of 15,507 deaths attributed to suicide. Of these, 12,072 (1.21% proportional mortality) were male, and 3,431 (0.43% proportional mortality) were female, indicating a substantial gender disparity in suicide rates. The mortality rates from suicide per 100,000 inhabitants varied significantly by age group: 0.7 for males and 0.9 for females in the 05 to 14 years age group; 9.3 for males and 4.5 for females in the 15 to 19 years group; 14.6 for males and 3.9 for females in the 20 to 29 years group; 14.9 for males and 3.8 for females in the 30 to 49 years group; 15.4 for males and 3.8 for females in the 50 to 69 years group; and 18.1 for males and 2.9 for females aged 70 years and older. Notably, among the leading causes of death, suicide ranked 11th for the 05 to 14 years age group (3.41%), 3rd for the 15 to 19 years age group (6.90%), and 4th for the 20 to 29 years age group (5.56%). These figures underscore the significant impact of suicide on young populations.

**Conclusions:**

The high mortality rate from suicide in Brazil underscores the urgent need for public health policies focused on suicide prevention. Effective interventions should include mental health support, community outreach programs, and increased awareness campaigns aimed at reducing the stigma around mental health issues. By addressing the underlying social and economic factors contributing to suicide, Brazil can improve health outcomes and enhance the quality of life for at-risk populations.

**Disclosure of Interest:**

None Declared

